# Lignin valorization: lignin nanoparticles as high-value bio-additive for multifunctional nanocomposites

**DOI:** 10.1186/s13068-017-0876-z

**Published:** 2017-07-24

**Authors:** Dong Tian, Jinguang Hu, Jie Bao, Richard P. Chandra, Jack N. Saddler, Canhui Lu

**Affiliations:** 1 State Key Laboratory of Polymer Materials Engineering, Polymer Research Institute of Sichuan University, Chengdu, 610065 China; 2 State Key Laboratory of Bioreactor Engineering, East China University of Science and Technology, 130 Meilong Road, Shanghai, 200237 China; 3 Forest Products Biotechnology/Bioenergy Group, Department of Wood Science, Faculty of Forestry, University of British Columbia, 2424 Main Mall, Vancouver, BC V6T 1Z4 Canada

**Keywords:** Lignin nanoparticles, UV-shielding, Antioxidant, Polymer nanocomposite, Biorefinery

## Abstract

**Background:**

Although conversion of low value but high-volume lignin by-product to its usable form is one of the determinant factors for building an economically feasible integrated lignocellulose biorefinery, it has been challenged by its structural complexity and inhomogeneity. We and others have shown that uniform lignin nanoparticles can be produced from a wide range of technical lignins, despite the varied lignocellulosic biomass and the pretreatment methods/conditions applied. This value-added nanostructure lignin enriched with multifunctional groups can be a promising versatile material platform for various downstream utilizations especially in the emerging nanocomposite fields.

**Results:**

Inspired by the story of successful production and application of nanocellulose biopolymer, two types of uniform lignin nanoparticles (LNPs) were prepared through self-assembling of deep eutectic solvent (DES) and ethanol-organosolv extracted technical lignins derived from a two-stage fractionation pretreatment approach, respectively. Both LPNs exhibited sphere morphology with unique core–shell nanostructure, where the DES–LNPs showed a more uniform particle size distribution. When incorporated into the traditional polymeric matrix such as poly(vinyl alcohol), these LPN products displayed great potential to formulate a transparent nanocomposite film with additional UV-shielding efficacy (reached ~80% at 400 nm with 4 wt% of LNPs) and antioxidant functionalities (reached ~160 μm mol Trolox g^−1^ with 4 wt% of LNPs). At the same time, the abundant phenolic hydroxyl groups on the shell of LNPs also provided good interfacial adhesion with PVA matrix through the formation of hydrogen bonding network, which further improved the mechanical and thermal performances of the fabricated LNPs/PVA nanocomposite films.

**Conclusions:**

Both LNPs are excellent candidates for producing multifunctional polymer nanocomposites using facile technical route. The prepared transparent and flexible LNPs/PVA composite films with high UV-shielding efficacy, antioxidant activity, and biocompatibility are promising in the advanced packaging field, which potentially provides an additional high-value lignin product stream to the lignocellulose biorefinery. This study could open the door for the production and application of novel LNPs in the nascent bioeconomy.

**Graphical abstract:**
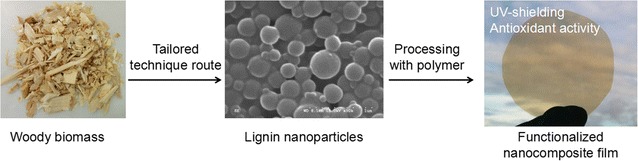
Lignin nanoparticle for transparent nanocomposite film with UV-shielding efficacy

**Electronic supplementary material:**

The online version of this article (doi:10.1186/s13068-017-0876-z) contains supplementary material, which is available to authorized users.

## Background

Conversion of major components (cellulose, hemicellulose, and lignin) of lignocellulosic biomass into usable platform is essential in the integrated biorefinery concept, which simplifies the subsequent production of fuels, chemicals, and materials [1, 2]. Although deconstructing of biomass carbohydrates into a valuable hexose/pentose sugar platform through biochemical approaches has been succeed for many years, the efficient utilization of lignin component has still been challenging [3, 4]. Both the traditional pulp and paper industry and the emerging cellulosic ethanol plant have been liberating a huge pile of technical lignins from lignocellulosic biomass; however, most of these lignins have been directly burned as the industrial “waste” for energy generation [4]. The high-value utilization of lignin via hydrocarbon fuel or aromatic polymer precursor production is attractive but still challenged by its structural complexity and inhomogeneity [5]. Recently, we and others have shown that the uniform lignin nanoparticles (LNPs) could be produced from a wide range of lignin by-products, regardless of their varied chemical structures [6–11]. These LNPs hold huge potential for downstream valorization due to their unique morphology and abundant multifunctional groups.

The nature of lignin is highly branched, three-dimensional polymer derived from three phenylpropane units (monolignols), namely, guaiacyl (G, conniferyl alcohol), syringyl (S, sinapyl alcohol), and p-hydroxyphenyl (H, p-coumaryl alcohol). When the prevalent solution-based self-assembly micellization process is employed to produce LNPs from the amphiphilic lignin fragments, the hydrophobic part of lignin (phenylpropanoid units) aggregates to form the micelle core in the solution, while the hydrophilic part of lignin (mainly phenolic and aliphatic hydroxyl groups) forms the micelle shell, simultaneously [6, 9]. Thus, the obtained LNPs exhibit unique core–shell nanostructure with abundant phenolic hydroxyl groups exposed on the shell of the LNPs. The greatly improved availability of phenolic hydroxyl groups on the shell of LNPs allows them to disperse well and stable in aqueous solution even for several months [7].

The production and application of functional polymer-based nanocomposites present new market opportunities for various bio-additives [12]. Although traditional inorganic nanomaterials could effectively endow the polymer nanocomposites with additional functionalities such as conductivity, antibacterial activity, flame resistance, etc., unexpected environmental and/or health problems occurs due to their poor biodegradability and biocompatibility [13]. In addition, some inorganic nanomaterials could also induce serious polymer matrix degradation. For example, when prevalent titanium dioxide and zinc oxide nanoparticles are used as UV-absorber additives, they catalyze the cleavage of polymer macromolecular chain due to their intrinsic photocatalytic activity [14–17]. Alternatively, LNPs which are produced from natural lignocellulosic biomass might be promising alternatives to those inorganic nanomaterials for producing functional polymer composites. Considering the outstanding UV shielding and antioxidant properties of the phenolic hydroxyl groups [18], LNPs might be suitable for producing functional protective nanocomposites by introducing functionalities to the polymer matrix while overcoming the above disadvantages from inorganic nanomaterials.

In the work reported here, we assessed the technical feasibility of valorizing lignin through producing LNPs/polymer nanocomposite films with both UV-shielding and antioxidant functionalities. Two technical lignins, DES and organosolv lignin isolated from steam pretreated hardwood poplar using a deep eutectic solvent (DES, an emerging solvent for biomass fractionation) and traditional ethanol organosolv, respectively [2, 19], were initially upgraded to their usable form of LNPs using prevalent micellization process. Then, the prepared LNPs were incorporated into the testing polymer poly(vinyl alcohol) (PVA), a biodegradable synthetic polymer material with wide commercial applications, to produce nanocomposite films via the facile solution-cast method. The overall performance of the resulting nanocomposite films including UV-shielding efficacy, antioxidant activity, and mechanical strength was systematically assessed. The possible interactions between two LNPs and PVA matrix were also comparatively evaluated. Results showed that both LNPs were great candidates for producing high-value functional polymer nanocomposites, while the organosolv LNPs with higher amount of phenolic hydroxyl groups exposed on the nanoparticle shell (assessed by quantitative ^31^P NMR) exhibited better overall performances than the DES LNPs [20]. We hope that the work reported here could open the door for the production and application of a wide range of novel LNP-based polymer nanocomposites.

## Results and discussion

### Synthesis and characterization of lignin nanoparticles

**Fig. 1:**
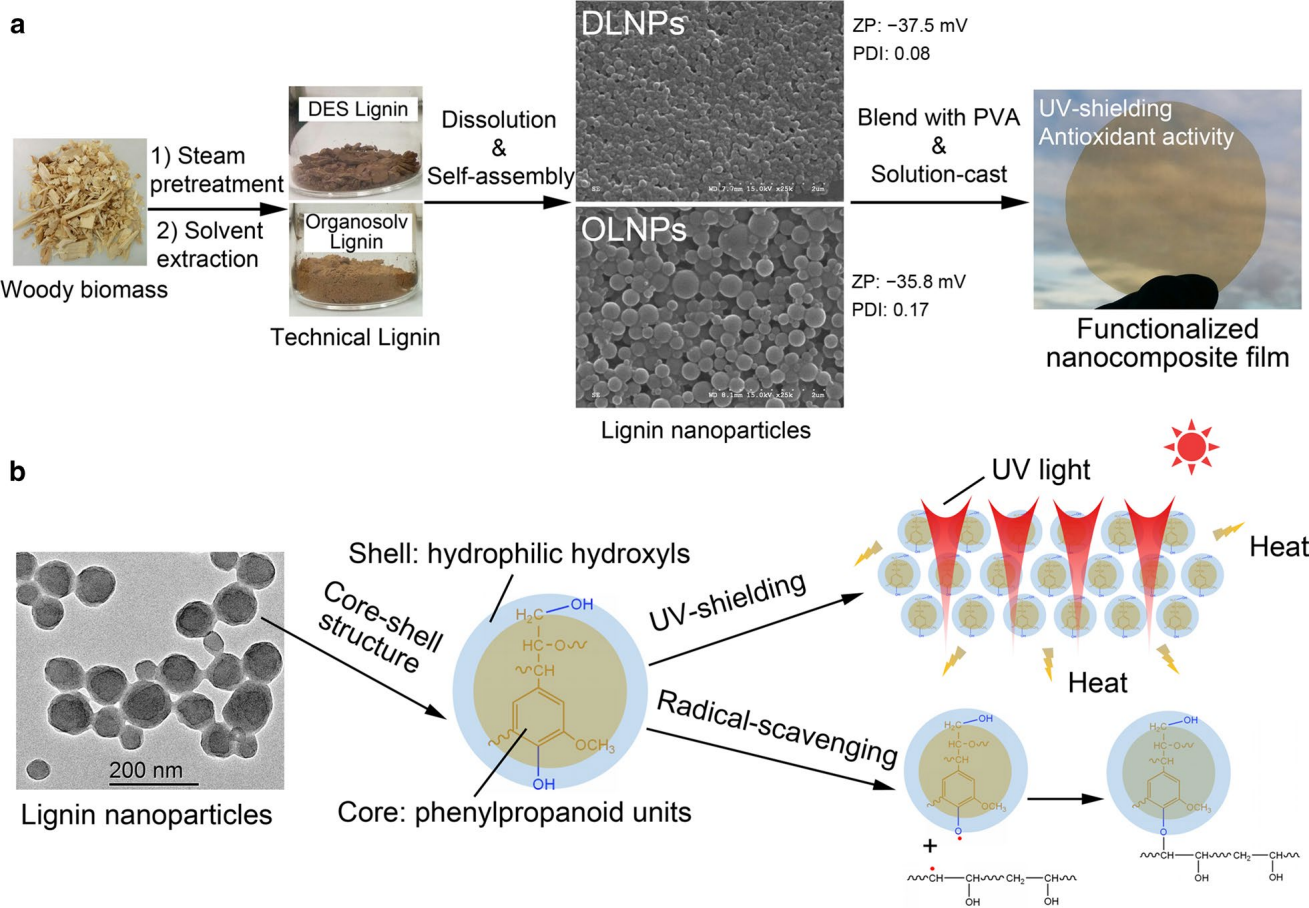
**a** Synthetic procedure to fabricate lignin nanoparticles and the lignin nanoparticles/PVA composite film. *ZP* Zeta-potential value, *PDI* polydispersity index. **b** Proposed mechanism for UV-shielding and antioxidant activity using lignin nanoparticles as the functional additive

To achieve full utilization of lignocellulosic biomass and easy integration of LNPs production into current biorefinery concept, a two-step pretreatment strategy, mild steam pretreatment followed by solvent extraction, was employed to produce DES and organosolv technical lignins from raw hardwood poplar while facilitating the conversion of cellulose/hemicellulose component to hexose/pentose sugar platform according to the previous reports [2, 21] (for the details of the fractionation pretreatment, see Additional file 1: Figure S1). The purity of the two technical lignins was higher than 98% according to HPLC analysis reported previously [9]. When these two technical lignins were dissolved in dimethylsulfoxide (2 mg mL^−1^) and subjected to micellization using dialysis, uniform lignin nanoparticle dispersions (referred as DLNPs and OLNPs, respectively) were obtained. Scan electron microscopy (SEM) and atomic force microscopy (AFM, Additional file 1: Figure S2) images showed that both lignin nanoparticles products had sphere morphological structure, while DLNPs had a more uniform particle size distribution (Fig. 1a). The core–shell structure of the two lignin nanoparticles was confirmed by high-resolution transmission electron microscopy (TEM) images (Additional file 1: Figure S3). The dark black color of the sphere particles indicates the core, while the grey color around it indicates the shell. The shell thickness was about 10–20 nm. When the dynamic properties of the two lignin nanoparticle dispersions were further analyzed by dynamic light scattering (DLS), the DLNPs gave an average particle size of 195 nm with a polydispersity index (PDI) of 0.08, while the OLNPs exhibited a similar average particle size (197 nm) but indeed a much higher PDI (0.17) (Fig. 1a). The zeta-potential value (also measured by DLS) of the lignin nanoparticle dispersion was −37.5 and −35.8 mV for DLNPs and OLNPs, respectively, which indicated a relative high stability of these two lignin nanoparticles in water [7]. The uniform particle size, regular-sphere structure, and high stability of these two lignin nanoparticles indicated that they might be promising candidates for the production of nanocomposite films with PVA polymer.

### UV shielding, antioxidant, and mechanical performance of the lignin nanoparticles/PVA composite films

**Fig. 2:**
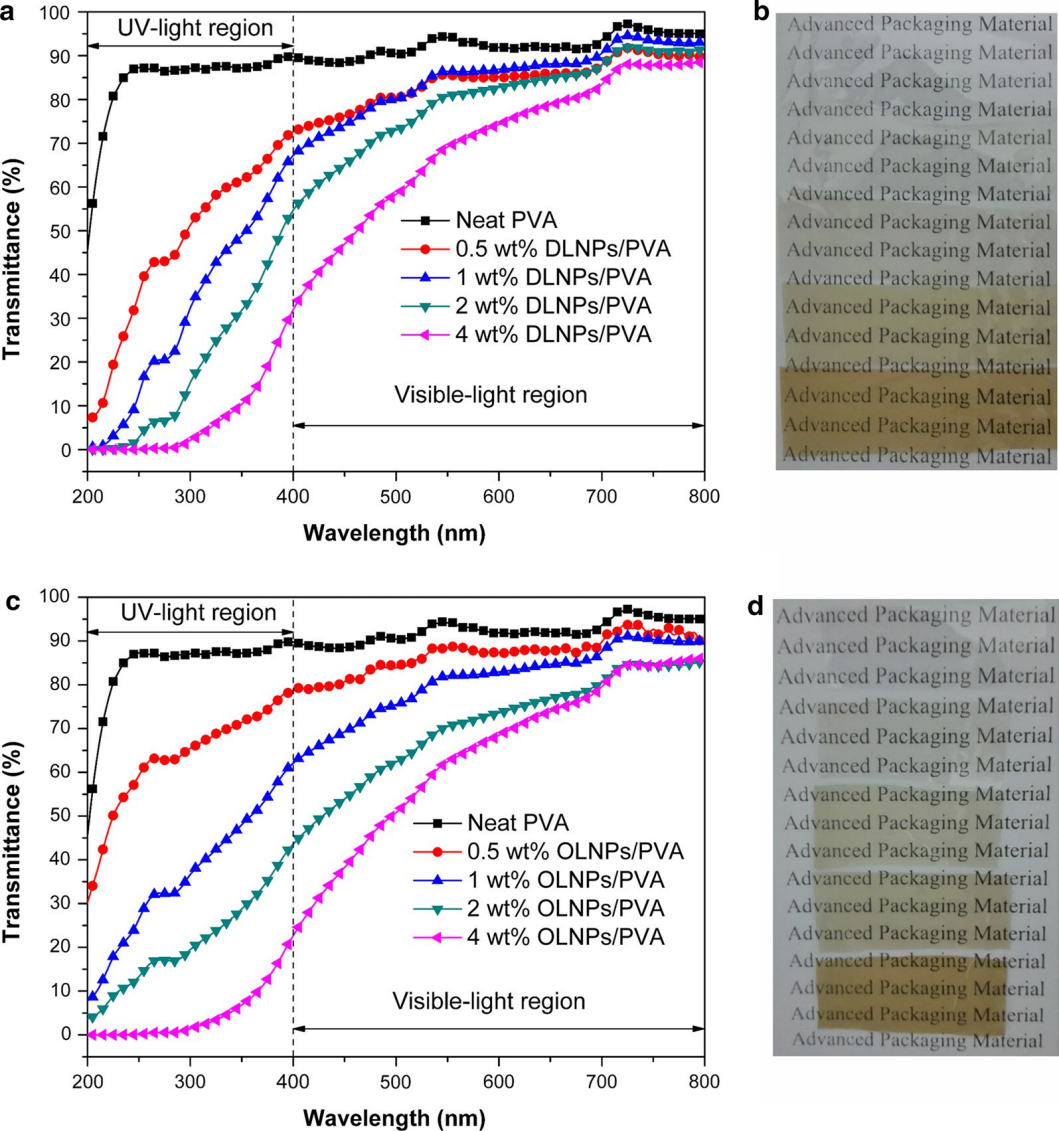
UV–Vis light transmittance spectra and digital photographs of **a** and **b** DLNPs/PVA and **c** and **d** OLNPs/PVA composite films with 0–4 wt% lignin nanoparticles. The digital photographs show the high optical transparency of the lignin nanoparticles/PVA composite films (from *top* to *bottom*, the content of lignin nanoparticles in the film was increasing from 0 to 4 wt%)

As expected, the lignin nanoparticles/PVA composites were easily prepared by a simple solution-cast method due to the good properties of these lignin nanoparticles as mentioned above (Fig. 1a). The influence of the content of lignin nanoparticles (0–4 wt% lignin nanoparticles based on the dry weight of PVA) on the UV shielding and antioxidant performances of the resulting composite films was first assessed. The related mechanism of UV shielding and antioxidant is proposed in Fig. 1b according to the previous reports [13, 15], and LNPs are suggested to block the ultraviolet light by absorbing its photon energy and further converting it to heat with the corresponding hydrophilic chromophores (mainly phenolic hydroxyl, carbonyl, and carboxyl groups) exposed on the particle shell. Then, the generated heat is gradually released out of the nanocomposite films without causing PVA degradation, while these phenolic hydroxyl groups could also easily quench active radicals through an electron transfer process [22, 23]. When all the prepared films were exposed to UV–Visible light with a wavelength from 200 to 800 nm (Fig. 2), it was apparent that the nanocomposite films could efficiently block the ultraviolet lights especially for UVB (280–315 nm) even with a low lignin nanoparticles content (0.5 wt%), while the neat PVA film (control) was almost transparent for all the testing ultraviolet lights (Fig. 2). Although further increase of lignin nanoparticles contents from 0.5 to 4 wt% resulted in an obvious improvement of the shielding efficacy for both UVB (280–315 nm) and UVA (315–400 nm), it slightly sacrificed the visible light transparency simultaneously (Fig. 2). In general, the OLNPs/PVA film exhibited higher shielding efficacy compared to the DLNPs/PVA film at the same lignin nanoparticles content, and it was also worth noting that the shielding efficacy of DLNPs/PVA and OLNPs/PVA nanocomposite films could reach nearly 100% for UVB with only 4% (w/w) lignin nanoparticles content (Fig. 2a, c). All these results indicated that the addition of lignin nanoparticle to the PVA film could provide an efficient UV block capacity without influencing its visible light transparency [11].

Further translating the UV–Visible transmittance spectra into Tauc’s plot with the frequency dependent absorption coefficient according to the previous reports provided additional information about the band structure and optical energy bandgap (*E*_g_) of the composite films [15, 24]. The principle of evaluating the optical property of the composites using *E*_g_ is that the photons with energy higher than the band-gap energy will be absorbed by the corresponding molecules in the composite [24]. When the *E*_g_ value of each composite film was calculated, it decreased with the increase of lignin nanoparticle content (3.81 − 2.49 eV for DLNPs/PVA and 3.54 − 1.96 eV for OLNPs/PVA, for the details of *E*_g_ calculation, see Additional file 1: Figure S4), indicating that more ultraviolet lights with wider wavelength range could be blocked. The *E*_g_ values also provided a fair compare of the overall optical performance among various UV-absorbing materials. The results showed that the UV-shielding efficacy of these two types of lignin nanoparticles was comparable to prevalent metal oxide nanoparticles and other emerging biopolymer nanoparticles such as polydopamine and melanin [13, 15, 25, 26].

**Fig. 3:**
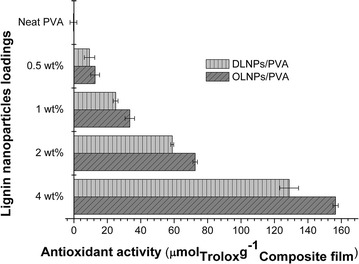
Antioxidant activities of lignin nanoparticles/poly(vinyl alcohol) (LNPs/PVA) composite films determined by TEAC assay

We further assessed the influence of lignin nanoparticles on the antioxidant properties of the prepared nanocomposite films using prevalent Trolox equivalent antioxidant capacity (TEAC) measurement, which employed stable 1,1-diphenyl-2-picrylhydrazyl (DPPH) as the testing radicals and Trolox as the internal standard to evaluate the radical-scavenging ability of the composites according to the previous reports (Fig. 3, and results were expressed as μmol Trolox per gram of composite film) [27]. As expected, the antioxidant activities of the films gradually increased with the increasing content of lignin nanoparticles, while OLNPs/PVA film exhibited higher antioxidant activity than DLNPs/PVA at the same lignin nanoparticles content, likely due to more available phenolic hydroxyl groups in the OLNPs [23, 28]. The antioxidant activity of the film was nearly zero for PVA, but dramatically increased to 129 (DLNPs/PVA) and 157 μm mol Trolox per gram composite (OLNPs/PVA) after incorporation of 4 wt% lignin nanoparticles. Previous reports have shown that the TEAC value of pure lignin and other natural phenolic compounds was ~500 μm mol Trolox g^−1^ [22, 27]. However, the work reported here showed that although the content of lignin nanoparticles in the composite films was only 4 wt%, their TEAC values could reach ~150 μm mol Trolox g^−1^ despite of different testing solvents employed. These results indicated that the prepared lignin nanoparticles had rather high radical-scavenging ability.

**Fig. 4:**
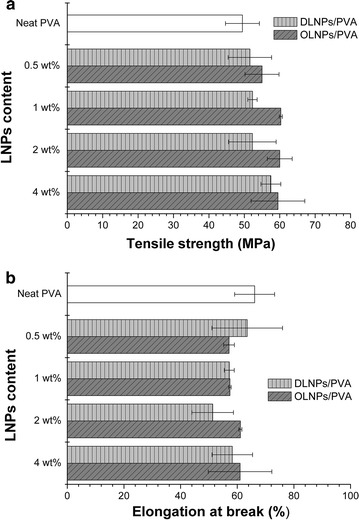
**a** Tensile strength and **b** elongation at break of neat PVA and LNPs/PVA composite films

Although bulk lignin could be directly blended with a polymer matrix using thermal extrusion/injection method to produce a composite, the poor interfacial binding between bulk lignin and the polymer matrix usually resulted in the deterioration of its mechanical performance [18]. The nanoeffects of lignin nanoparticles including increased surface area and good dispersion state potentially enhance their compatibility with polymer matrix; thus, the resulting composite might exhibit a better mechanical performance [8, 11]. When the mechanical properties of the above nanocomposites were further checked, there was indeed an increase instead of a deterioration of the tensile strength, indicating a certain reinforcement effect of lignin nanoparticles. The tensile strength of the composite films increased from 50 to ~55 MPa for DLNPs/PVA and to ~60 MPa for OLNPs/PVA while only slightly compromising its elongation performance simultaneously (Fig. 4). It seemed that apart from the intrinsic properties of lignin nanoparticles themselves, their interfacial adhesion and dispersion state in the PVA matrix also played an important role in determining the overall performances of the nanocomposites. Therefore, we next assessed the interactions between lignin nanoparticles and PVA matrix. As the composite films containing 4 wt% lignin nanoparticles had the best UV shielding, antioxidant, and mechanical performances, we selected them as the testing samples for the subsequent analysis.

### Interactions between lignin nanoparticles and PVA matrix

**Fig. 5:**
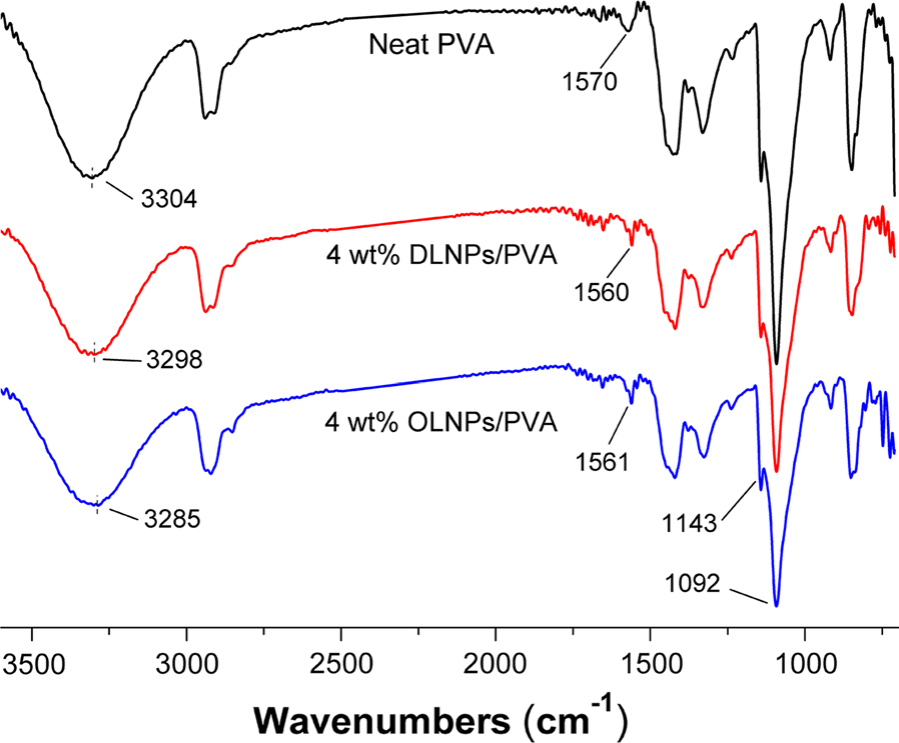
FTIR spectra of neat PVA and the composite films with 4 wt% lignin nanoparticles

As mentioned earlier, the shell of the lignin nanoparticles was mainly composed of hydrophilic hydroxyl groups, which could potentially form strong interactions with the hydroxyl groups of PVA [6]. When Fourier transform infrared spectroscopy (FTIR) analysis was conducted on the selected samples to confirm this hypothesis, it was shown that the –OH stretching band for the neat PVA shifted from 3304 cm^−1^ to a lower wavenumber of 3298 cm^−1^ (DLNPs/PVA) and 3285 cm^−1^ (OLNPs/PVA), respectively, upon incorporating with 4 wt% lignin nanoparticles, indicating that hydrogen bonds were formed between the PVA and the lignin nanoparticles (Fig. 5) [29]. In addition, the FTIR spectrum of neat PVA exhibited a strong stretching vibrational band of carbon–carbon double bonds at 1570 cm^−1^, which was likely resulted by the radical-induced degradation of PVA macromolecular chains, since the PVA solution was prepared under heating at an open atmosphere (Fig. 5) [30]. However, this absorption peak became much narrower and also shifted to lower wavenumbers (1560 cm^−1^ for DLNPs/PVA and 1561 cm^−1^ for OLNPs/PVA, respectively) when lignin nanoparticles were added, indicating that lignin nanoparticles could stabilize the PVA macromolecular structure through trapping the generated free radicals [15]. The carbon–carbon double bonds in the composite films might be negatively charged by lignin nanoparticles; thus, their FTIR spectra exhibited such a wavenumber shift according to the previous reports [15, 30]. The above analysis suggested that the lignin nanoparticles were able to interact with the PVA macromolecular chains through hydrogen bonding and radical-scavenging reactions.

**Fig. 6:**
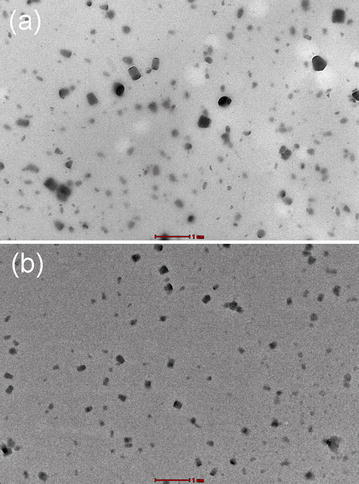
TEM images of the cross section of **a** DLNPs/PVA and **b** OLNPs/PVA composite film with 4 wt% lignin nanoparticles

The good interfacial adhesion between lignin nanoparticles and PVA matrix was further evidenced by transmission electron microscopy (TEM) observations, as shown in Fig. 6. Due to the good dispersion state of the lignin nanoparticles in water, the resulting nanocomposite films exhibited well-defined sea-island structure, where lignin nanoparticles were dispersed at nanoscale without aggregation. The morphological structure of the lignin nanoparticles was slightly changed after incorporation into the PVA matrix, but still remained their sphere-like shape (Figs. 1, 6), suggesting that the shell of lignin nanoparticles (composed of hydroxyl groups) indeed interacted with PVA as hypothesized earlier. These strong interactions were likely the driving force in improving the mechanical performance of the composite films. In addition, the good dispersion state of lignin nanoparticles was also responsible for the excellent UV shielding and antioxidant performance of the composite films.

### Influence of lignin nanoparticles on the crystalline and thermal properties of the composite films

It is acknowledged that the nanofiller itself and its interactions with the polymer matrix can affect the crystalline and thermal properties of the resulting composites, which also play an important role in determining the downstream processability and usability of the composites [13]. Thus, crystalline and thermal analysis were subsequently carried out with the selected sample films using prevalent techniques [differential scanning calorimetry (DSC), X-ray diffraction (XRD), and thermogravimetric analysis (TGA)] and the crucial results are summarized in Table 1 (for detail information of the results, see Additional file 1: Figures S5–S7). It was shown that the melting point (*T*_m_) of the composite films was unchanged compared to the neat PVA film, but the degree of crystallinity (*X*_c_) was decreased from 21.7 to 19.2% for DLNPs/PVA and to 15.6% for OLNPs/PVA, respectively. For neat PVA, the macromolecular chains regularly stacked together to form crystalline regions, where the extensively existed intra- and intermolecular hydrogen bonding network could further enhance its crystallization [31], whereas incorporation of the amorphous lignin nanoparticles disrupted this hydrogen bonding network and new hydrogen bonds were formed between lignin nanoparticles and PVA matrix (Fig. 5), leading to less crystalline regions formed in the composite films (Table 1) [29]. In contrast to other biopolymer nanoparticles that they could facilitate the crystallization process through the nucleation effect [13], lignin nanoparticles showed limited contribution to the PVA crystal formation and growth, even though they were compatible with PVA matrix. As evidenced by XRD analysis, the crystalline structure of the composites was nearly the same as that of neat PVA film (Additional file 1: Figure S6).

**Table 1:** Crystalline and thermal properties of neat PVA and lignin nanoparticles/PVA composite films

Sample	*T*_m_ (°C)	Δ*H*_m_ (J g^−1^)	*X*_c_ (%)	*τ* (nm)	*T*_i_ (°C)	*T*_p_ (°C)
Neat PVA	229	34.9	21.7	3.3	247	270
4 wt% DLNPs/PVA	229	29.7	19.2	3.3	253	274
4 wt% OLNPs/PVA	228	24.1	15.6	3.6	254	278

*T*_*m*_ melting point, *ΔH*_*m*_ the heat of fusion, *X*_*c*_ degree of crystallinity, *τ* crystal size, and *T*_*i*_ and *T*_*p*_ initial and maximum decomposition temperature, respectively

TGA results show that the thermal stability of the composite films was slightly improved when lignin nanoparticles were incorporated. Neat PVA film exhibited an initial decomposition temperature (*T*_i_) of 247 °C and a maximum decomposition temperature (*T*_p_) of 270 °C, respectively, corresponding to the elimination of side hydroxyl groups and the partial chain-scission process, where a considerable amount of free radicals was generated [32]. It was likely that the incorporated lignin nanoparticles could trap these radicals and, therefore, retard the decomposition of the composites. As shown in Table 1, both DLNPs/PVA and OLNPs/PVA composites exhibited higher *T*_i_ and *T*_p_. Such thermal stability improvement for other PVA-based nanocomposites has also been reported, which contain similar phenolic compound nanofillers such as melanin, polydopamine, and Kraft nanolignin particles prepared through high shear homogenization [10, 13, 33].

### Phenolic substructures of the lignin nanoparticles

It was interesting that although DLNPs and OLNPs were derived from the same biomass substrate, both of which exhibited similar particle size (about 200 nm) and sphere morphology, OLNPs showed a better performance in enhancing the overall protective, mechanical, and thermal properties of the prepared composite films according to the above results. We notice that the phenolic hydroxyls within the lignin nanoparticles are the main responsible functional groups that influence the overall properties of the nanocomposites, which encouraged us to further look at the phenolic substructures of these two lignin nanoparticles using emerging quantitative ^31^P NMR technic according to previously reported procedures [20]. In this method, an internal standard and the lignin sample are suitably phosphitylated with the phosphorous reagent, and then, all the phosphorus-tagged hydroxyl groups belonging to lignin including phenolic, aliphatic, and carboxylic hydroxyls could be readily quantified by ^31^P NMR spectroscopy. When the contents and locations of the hydroxyl groups in these two lignin nanoparticles were determined and compared (Table 2, for the ^31^P NMR spectra, see Additional file 1: Figure S8), it was apparent that OLNPs indeed had a higher amount of total phenolic hydroxyl groups (3.37 mmol g^−1^) compared to DLPNs (2.44 mmol g^−1^) as expected before. Organosolv extraction employing a much aggressive extraction solvent (ethanol–water) with sulphuric acid as the catalyst tended to extensively cleavage the β–O–4′ linkages in the biomass lignin thus forms a large amount of free phenolic hydroxyl groups [23]. Meanwhile, the acid-catalyzed condensation between the aromatic active sites and the generated free radicals resulted in an increased content of syringyl hydroxyl groups (2.03 mmol g^−1^) [2]. On the contrary, DES lignin extracted by lactic acid–betaine solvent system at milder conditions likely underwent less fragmentation and condensation; therefore, the resulting DLNPs showed less content of total phenolic hydroxyl groups and condensed aromatics (Table 2) [21]. To conclude, the higher content of total phenolic hydroxyl groups in the lignin nanoparticles not only enabled the resulting composite film with higher UV-shielding and radical-scavenging ability, but also provided stronger interactions between the lignin nanoparticles and the PVA matrix.

**Table 2:** Contents (mmol g^−1^) and locations of hydroxyl groups in these two lignin nanoparticles as determined by quantitative ^31^P NMR spectroscopy

Sample	DLNPs	OLNPs
Aliphatic–OH	2.40	1.04
Syringyl–OH	1.31	2.03
Guaiacyl–OH	0.90	1.20
*p*-hydroxyphenyl–OH	0.23	0.15
Carboxylic–OH	0.18	0.09
Total phenolic–OH	2.44	3.37

## Conclusions

Lignin nanoparticles are good candidates for next-generation functional nanocomposites. In addition to blocking ultraviolet light and scavenging free radicals, the enriched phenolic hydroxyl groups on the shell of the lignin nanoparticles also provide good interfacial adhesion with poly(vinyl alcohol). It is also shown that the phenolic substructures of the lignin nanoparticles, which are determined by the employed extraction/pretreatment technique routes, significantly influence the overall properties of the downstream nanocomposite films. Further conducting a hydrophobic modification on these lignin nanoparticles potentially enables them to be compatible with nonpolar polymer matrix such as polyethylene and polypropylene; therefore, their applications could be greatly extended.

## Experimental

### Fabrication and characterization of lignin nanoparticles

A mild steam pretreatment was conducted with raw hardwood poplar to pre-extract hemicellulose while facilitating the subsequent lignin extraction according to method previously reported [34]. The DES formulated by a certain amount of lactic acid and betaine was selected as the extraction solvent, since it showed quite high lignin solubility among all the assessed DESs as reported previously [21]. DES lignin extraction was carried out at atmospheric pressure on a hot plate equipped with a digital controller and magnetic stirring. One gram of steam pretreated poplar biomass (dry matter) and 20 g of DES were transferred to a 100 mL conical flask and heated at 130 °C for 3 h with continuous stirring. The reaction mixture was then cooled to about 80 °C, after which acetone/water mixture (50/50 by volume) was added to wash the DES and lignin away from the obtained cellulose-rich pulp by vacuum filtration. DES lignin was precipitated by evaporating of acetone from the washes and washed with distilled water three times. Organosolv lignin was obtained using ethanol/water (50/50 by wt, 1% H_2_SO_4_ as the catalyst) as the extraction solvent with the optimized conditions according to our previous report [2]. 100 g (on a dry matter basis) of steam pretreated poplar biomass was cooked at 170 °C for 1 h at a liquid-to-solid ratio of 7:1 using a four-vessel (2 L each) rotating digester (Aurora Products, Savona, BC, Canada). At the end of the extraction period, the vessel was cooled to room temperature in a water bath. Then, the liquid fraction was collected by vacuum filtration. Organosolv lignin was precipitated by adding ten times of hot water to the filtrate, and then, the collected lignin was washed with distilled water. Both these two obtained technical lignins were dried in a vacuum oven for further use.

The prevalent dialysis method was employed in this study to fabricate lignin nanoparticles. Briefly, 400 mg of the technical lignin was dissolved in 200 mL of dimethylsulfoxide [DMSO, its Hildebrand solubility parameter (*δ* value) was close to various types of lignin] to form a homogenous solution [35]. Then, the resulting lignin solution was introduced into a dialysis tube (Spectra/Por^®^ 2 Standard RC Dry Dialysis Tubing, 12–14 kDa, Spectrum Labs, USA). The dialysis was conducted in excess of tap water (periodically replaced) and stopped until no DMSO trace was checked in the waste water. Finally, the obtained lignin nanoparticle dispersion was stored in the refrigerator (4 °C) for further characterization after adjusting its concentration to 4 mg mL^−1^ by evaporating excess water.

Scanning electron microscopy (SEM) images of the lignin nanoparticles were taken using a JEOL JSM-5600 SEM (Japan) with the freeze-dried sample. Prior to imaging, the sample was sputter-coated with Pd–Au alloy to build up the charge on the surface.

The particle size, particle size distribution, and zeta potential of the lignin nanoparticle dispersions were measured with a Malvern Zetasizer Nano-ZS90 Instrument.

### Preparation and characterization of lignin nanoparticles/PVA composite films

The composite films were prepared using facile solution-cast method. First, 10 g of PVA (Product No. 563900, Sigma-Aldrich) was dissolved in 190 g of water with heating (90 °C) for 2 h to make a 5 wt% PVA solution. Then, the required amount of lignin nanoparticle dispersion and PVA solution was mixed and sonicated for 1 h. After that, the homogeneous mixture was degased and poured onto a polished glass plate. The composite films were obtained by evaporating the water from the gel-like films at room temperature and dried at 60 °C in the oven. The resulting composite films were quite uniform with an average thickness of about 30 μm.

The UV-shielding performance and optical transparency of the composite films were measured on a UV–Visible spectrophotometer. The free-radical-scavenging activity was evaluated using TEAC assay according to the method previously reported with some modifications [22, 27]. Briefly, 7.4 mg of Diphenyl-1-picrylhydrazyl (DPPH, Product No. D9132, Sigma-Aldrich) was dissolved in 100 mL of methanol to obtain an absorbance of 1.8 at 520 nm. Each composite sample (30 mg) was dissolved in 5 mL of DMSO and stirred for 3 h. Then, 1 ml of the fresh DPPH solution was mixed with 0.2 µL of the sample solution and incubated for 1 h at 30 °C. The absorption of the reacted mixture was immediately measured at 520 nm. Trolox (Product No. 238813, Sigma-Aldrich) solutions in DMSO at various concentrations (0.1–1 mmol L^−1^) were used for calibration. The results were expressed as µmol equivalents of Trolox per gram of the composite film.

Mechanical properties were measured using Instron 5567 Universal Testing Machine at a crosshead speed of 50 mm min^−1^. Five specimens of each sample were tested and the averaged results were presented.

Fourier transform infrared (FTIR) spectra were conducted by a Nicolet 560 spectrophotometer (USA). Transmission electron microscopy (TEM) was performed using a transmission electron microscope (JEOL JEM-100CX, Japan). The sample was prepared by ultrathin section before imaging. Differential scanning calorimetry (DSC) analysis was conducted on a NETZSH 204 DSC differential scanning calorimeter under a flowing N_2_ with the heating scan from ambient temperature to 250 °C at a heating rate of 10 °C min^−1^. The degree of crystallinity of PVA was calculated based on the following equation [31]:

$${\text{X}}_{\text{c}} = \frac{{{\Delta}{{H}}_{{{m}}} }}{{{{w}}{\Delta }{{H}}_{{{{m}}{\text{o}}}} }}$$

where *w* is the weight fraction of PVA matrix in the composite film, Δ*H*_m_ is the heat of fusion of the composite, and Δ*H*_mo_ is the heat of fusion of 100% crystalline PVA (161 J g^−1^). X-ray diffraction (XRD) patterns were collected on a Philips Analytical X’Pert X-diffractometer (Philips Co., Netherlands), using Cu–Ka radiation (*λ* = 0.1540 nm) at an accelerating voltage of 40 kV and the current of 40 mA. The data were collected from 2*θ* = 5–60° with a step interval of 0.03°. The crystallite size was calculated using the Scherrer equation [31]:

$$\tau = \frac{K\lambda }{{\beta \;{ \cos }\theta }}$$

where *K* is a constant (0.94), *β* is the full-width at half-maximum in radians and *θ* is the position of the peak (half of the plotted 2*θ* value).

Thermal stability was measured on a TG209 F1 instrument (NETZSCH Co., Germany). About 5–8 mg of the composite sample was heated in a platinum crucible from room temperature to 600 °C at a heating rate of 20 °C min^−1^ under nitrogen atmosphere.

### Determine the content of hydroxyl groups of lignin nanoparticles using ^31^P NMR spectroscopy

^31^P NMR analysis was performed following the reported procedure [27]. An accurately weighed amount of lignin (20 mg) was dissolved in 500 μL of an anhydrous pyridine and deuterated chloroform mixture (1.6:1, v/v) with stirring. The anhydrous pyridine was purified by Soxlet’s extraction and dewatered with molecular sieve prior to use. 100 μL of cyclohexanol (10.85 mg mL^−1^ in anhydrous pyridine and deuterated chloroform 1.6:1, v/v) and 100 μL of chromium(III) acetylacetonate solution (5 mg mL^−1^ in anhydrous pyridine and deuterated chloroform 1.6:1, v/v) were further added as an internal standard and relaxation reagent, respectively. The mixture was reacted with 100 μL of phosphitylating reagent (2-chloro-4,4,5,5-tetramethyl-1,3,2-dioxaphospholane, TMDP, Product No. 447536, Sigma-Aldrich) and transferred into a 5 mm NMR tube. The NMR spectra of freshly prepared samples were acquired immediately at room temperature on a Bruker AV II 600 MHz spectrometer equipped with a QNP cryoprobe. Chemical shifts were calibrated relative to the phosphitylation product of TMDP with water (sample moisture), which gave a sharp and stable signal at 132.2 ppm.

## Additional file

**Additional file 1: Figure S1.** Material balance of each crucial step during the two-stage fractionation pretreatment approach. The tailored two-stage pretreatment could greatly enhance the enzymatic hydrolyzability of cellulose fraction while producing a usable lignin fraction for further valorization. **Figure S2.** AFM images of (a) and (b) DLNPs and (c) and (d) OLNPs. **Figure S3.** High-resolution TEM images of (a) DLNPs and (b) OLNPs. **Figure S4.** Translation of the UV–Visible transmittance spectra into Tauc’s plots to calculate the optical energy bandgap (E_g_) of each nanocomposite film (a) DLNPs/PVA, (b) OLNPs/PVA. **Figure S5.** Differential scanning calorimetry (DSC) curves of heating scans for neat PVA and 4 wt% lignin nanoparticles/PVA composite films. **Figure S6.** X-ray diffraction (XRD) patterns of neat PVA and 4 wt% lignin nanoparticles/PVA composite films. **Figure S7.** Thermal gravity (TG) and Differential thermal gravity (DTG) curves of neat PVA and 4 wt% lignin nanoparticles/PVA composite films. **Figure S8.** Quantitative ^31^P NMR spectra of these two lignin nanoparticles tagged with the phosphorous reagent using cyclohexanol as internal standard

## Abbreviations

DES: deep eutectic solvent
LNPs: lignin nanoparticles
DLNPs: deep eutectic solvent lignin nanoparticles
OLNPs: organosolv lignin nanoparticles
UV: ultraviolet light
PVA: poly(vinyl alcohol)
DPPH: 1,1-diphenyl-2-picrylhydrazyl
TEAC: trolox equivalent antioxidant capacity
XRD: X-ray diffraction patterns
CrI: crystallinity index
FTIR: Fourier transform infrared spectroscopy
TEM: transmission electron microscopy
SEM: scanning electron microscopy
DMSO: dimethylsulfoxide
DSC: differential scanning calorimetry
TGA: thermogravimetric analysis
HPLC: high-performance liquid chromatography
