# Supplementary material for: Lignin valorization: lignin nanoparticles as high-value bio-additive for multifunctional nanocomposites
Source: Biotechnol Biofuels. 2017 Jul 24;10:192. doi: 10.1186/s13068-017-0876-z (PMC5525242; doi:10.1186/s13068-017-0876-z)
Supplement: Supplementary file 1 — Additional file 1: Figure S1. Material balance of each crucial step during the two-stage fractionation pretreatment approach. The tailored two-stage pretreatment could greatly enhance the enzymatic hydrolyzability of cellulose fraction while producing a usable lignin fraction for further valorization. Figure S2. AFM images of (a) and (b) DLNPs and (c) and (d) OLNPs. Figure S3. High-resolution TEM images of (a) DLNPs and (b) OLNPs. Figure S4. Translation of the UV–Visible transmittance spectra into Tauc’s plots to calculate the optical energy bandgap (Eg) of each nanocomposite film (a) DLNPs/PVA, (b) OLNPs/PVA. Figure S5. Differential scanning calorimetry (DSC) curves of heating scans for neat PVA and 4 wt% lignin nanoparticles/PVA composite films. Figure S6. X-ray diffraction (XRD) patterns of neat PVA and 4 wt% lignin nanoparticles/PVA composite films. Figure S7. Thermal gravity (TG) and Differential thermal gravity (DTG) curves of neat PVA and 4 wt% lignin nanoparticles/PVA composite films. Figure S8. Quantitative 31P NMR spectra of these two lignin nanoparticles tagged with the phosphorous reagent using cyclohexanol as internal standard [file 13068_2017_876_MOESM1_ESM.docx]

Electronic Supplementary Information for

**Lignin valorization: lignin nanoparticles as high-value additive for multifunctional biocomposites**

Dong Tian^1,2,3^, Jinguang Hu^2,3^*, Jie Bao^2^, Richard P. Chandra^3^, Jack N. Saddler^3^, Canhui Lu^1^*

^1^State Key Laboratory of Polymer Materials Engineering, Polymer Research Institute of Sichuan University, Chengdu 610065, China

^2^State Key Laboratory of Bioreactor Engineering, East China University of Science and Technology, 130 Meilong Road, Shanghai 200237, China

^3^Forest Products Biotechnology/Bioenergy Group, Department of Wood Science, Faculty of Forestry, University of British Columbia, 2424 Main Mall, Vancouver, British Columbia V6T 1Z4, Canada

*Corresponding authors:

Jinguang Hu: jinguang@mail.ubc.ca

Canhui Lu: canhuilu@scu.edu.cn

Contents

Eight pages (including cover sheet)

Eight figures (Figure S1–8)


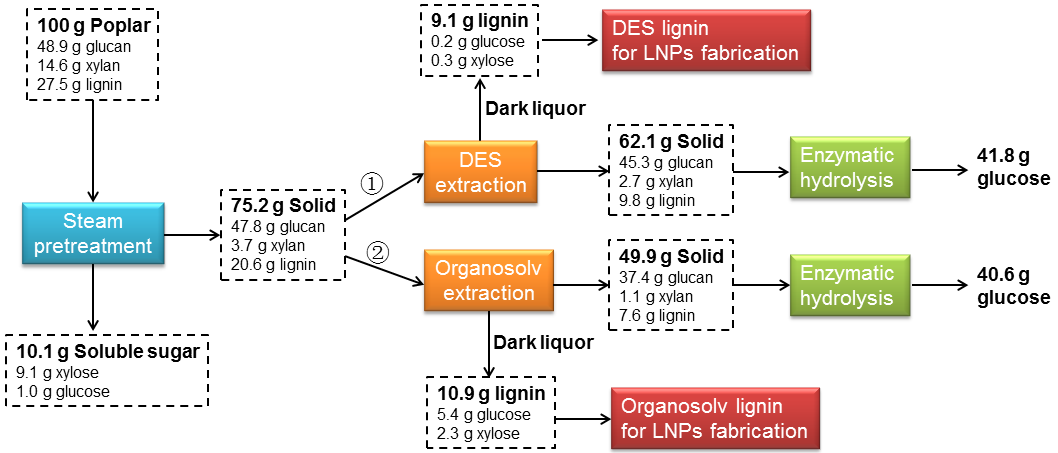


**Figure S1** Material balance of each crucial step during the two-stage fractionation pretreatment approach. The tailored two-stage pretreatment could greatly enhance the enzymatic hydrolyzability of cellulose fraction while producing a usable lignin fraction for further valorization.

Steam retreatment conditions: 190 ^o^C, 9.9 min, 0.7 wt% H_2_SO_4_ as the catalyst to enhance hemicellulos solubilization. DES: a deep eutectic solvent formulated by mixing lactic acid and betaine with a malor ratio of 2.5 to 1. DES extraction conditions: 130 ^o^C, 3 h, liquid to solid ratio, 20: 1. Organosolv: ethanol/water, 50/50 by wt, 1% H_2_SO_4_. Organosolv extraction conditions: 170 ^o^C, 1 h, liquid to solid ratio, 7: 1. Enzymatic hydrolysis conditions: solids loading, 2% w/v, enzyme loading, 8 mg _enzyme_ g^-1^_glucan_, Cellic CTec3.


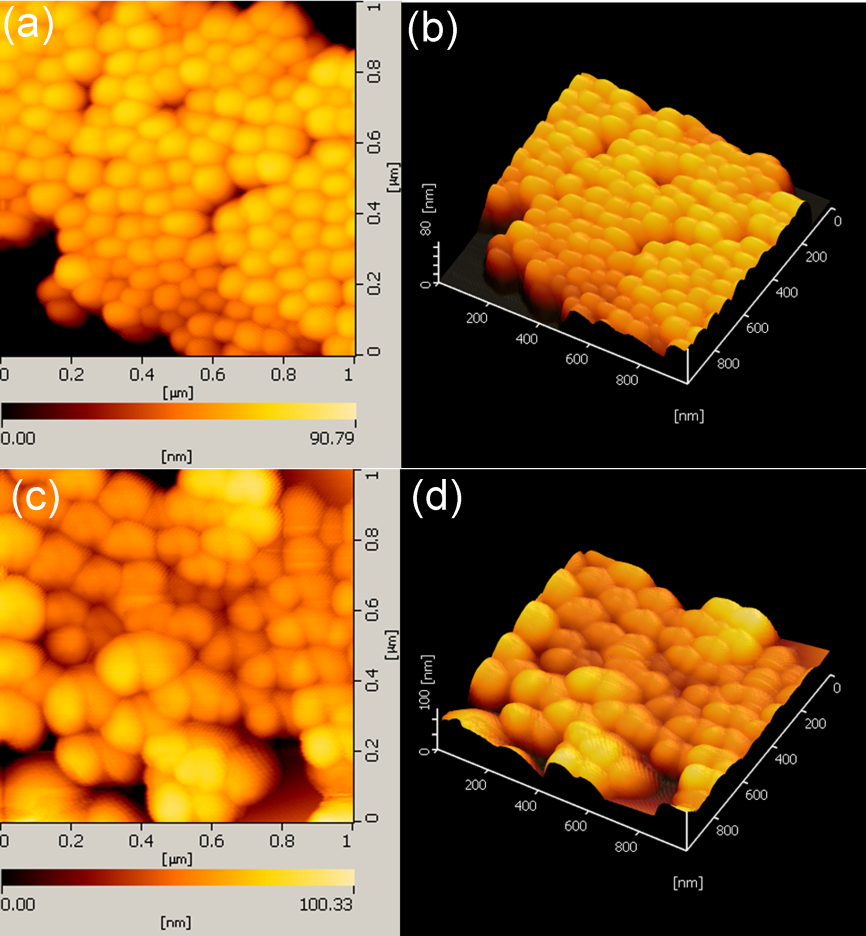


**Figure S2** AFM images of (a) (b) DLNPs and (c) (d) OLNPs.





**Figure S3** High-resolution TEM images of (a) DLNPs and (b) OLNPs.





**Figure S4** Translation of the UV-visible transmittance spectra into Tauc’s plots to calculate the optical energy band gap (E_g_) of each nanocmoposite film (a) DLNPs/PVA, (b) OLNPs/PVA.

The E_g_ of the composite film was calculated by Tauc’s expression.

$$\alpha h\upsilon=\beta\left( h\upsilon-E_{g} \right)^{n}$$

where α was the absorption coefficient, *h* was the Planck’s constant, υ was frequency of the incident photons, β was a constant and n was an empirical index (n=2 in this case). The absorption coefficient α was determined according to the following equation.

$$\alpha=\frac{2.303}{d}\log\frac{I}{I_{o}}=\left( \frac{2.303}{d} \right)A$$

where *d* was the thickness of the film and *A* was the absorbance. A curve was obtained by plotting (α*h*υ)^1/2^ against *h*υ and extrapolation of this linear portion of the curve to zero absorption gave the E_g_ of the testing film.





**Figure S5** Differential scanning calorimetry (DSC) curves of heating scans for neat PVA and 4 wt% lignin nanoparticles/PVA composite films.

**

**

**Figure S6** X-ray diffraction (XRD) patterns of neat PVA and 4 wt% lignin nanoparticles/PVA composite films.

**

**

**Figure S7** Thermal gravity (TG) and Differential thermal gravity (DTG) curves of neat PVA and 4 wt% lignin nanoparticles/PVA composite films.





**Figure S8** Quantitative ^31^P NMR spectra of these two lignin nanoparticles tagged with the phosphorous reagent using cyclohexanol as internal standard.
